# Prevalence of bacterial contamination of touchscreens and posterior surfaces of smartphones owned by healthcare workers: a cross-sectional study

**DOI:** 10.1186/s12879-021-06379-y

**Published:** 2021-07-13

**Authors:** Akira Kuriyama, Hiroyuki Fujii, Aki Hotta, Rina Asanuma, Hiromasa Irie

**Affiliations:** 1 Emergency and Critical Care Center, Kurashiki Central Hospital, Okayama, Japan; 2 Department of Clinical Laboratory, Kurashiki Central Hospital, Okayama, Japan; 3 Intensive Care Unit, Kurashiki Central Hospital, Okayama, Japan; 4 Emergency Intensive Care Unit, Kurashiki Central Hospital, Okayama, Japan; 5 Department of Anesthesiology, Kurashiki Central Hospital, Okayama, Japan

**Keywords:** Cell phone, Equipment contamination, Smartphone

## Abstract

**Background:**

Mobile phones used by healthcare workers (HCWs) are contaminated with bacteria, but the posterior surface of smartphones has rarely been studied. The aim of this study was to compare the prevalence of microbial contamination of touchscreens and posterior surfaces of smartphones owned by HCWs.

**Methods:**

A cross-sectional study of smartphones used by HCWs employed at two intensive care units at a Japanese tertiary care hospital was performed. Bacteria on each surface of the smartphones were isolated separately. The primary outcomes were the prevalence of microbial contamination on each surface of smartphones and associated bacterial species. Fisher’s exact test was used to compare dichotomous outcomes.

**Results:**

Eighty-four HCWs participated in this study. The touchscreen and posterior surface were contaminated in 27 (32.1%) and 39 (46.4%) smartphones, respectively, indicating that the posterior surface was more frequently contaminated (*p* = 0.041). *Bacillus* species and coagulase-negative staphylococci were isolated from each surface of the smartphones.

**Conclusions:**

The posterior surface of a smartphone was more significantly contaminated with bacteria than the touchscreen, regardless of having a cover. Therefore, routine cleaning of the posterior surface of a smartphone is recommended.

## Introduction

Smartphone use is globally on the rise. Approximately 5.2 billion people, 67% of the global population, subscribed to mobile services by the end of 2019, and this proportion is expected to increase to 70% by 2025 [1]. Similarly, the roles of smartphones in healthcare settings have expanded. Smartphones are equipped with useful applications and allow clinicians to seek timely information on-site [2]. A multicenter survey in the United Kingdom in 2015 suggested that more than 90% of physicians found smartphones useful and used applications in their clinical practice [3]. Furthermore, communications via applications equipped in mobile phones are reported to be effective in preventive medicine [4] and treatment of chronic conditions [5]. Given the coronavirus disease 2019 (COVID-19) pandemic, an increasing clinical role of smartphones is expected [6–10].

Previous studies have suggested that mobile phones, whether keypad mobile phones or smartphones, used by healthcare workers (HCWs) are contaminated with bacteria, with prevalence rates ranging from 10 to 100% [11–13]. Although direct evidence is missing, several studies suggested that bacteria might have disseminated from HCWs’ smartphones to their hands or vice versa [14–21]. There is an anticipated increased chance of bacterial transmission from smartphones to the healthcare environment as more smartphones are used in healthcare settings.

Many previous studies of microbial contamination of mobile phones focused mainly on keypad mobile phones and the touchscreen of smartphones [11–13]. Posterior surfaces of smartphones are less frequently cleaned because they are often covered or contained in cases and are not considered high-touch areas due to their limited roles relative to the touchscreen. Thus, posterior surfaces of smartphones can serve as an example of the usual bacterial reservoirs as seen in touchscreens. To the best of our knowledge, few studies have specifically differentiated the touchscreen and posterior surface of smartphones and reported their microbial contamination separately.

Thus, this study was performed to compare the prevalence of microbial contamination of both surfaces of smartphones owned by HCWs and to investigate factors associated with microbial contamination of the posterior surfaces of smartphones.

## Methods

### Study design and setting

This was a cross-sectional study conducted in intensive care units (ICUs) in a Japanese hospital in March 2017. The hospital is a 1172-bed, tertiary care center located in western Japan that provides care for approximately 800,000 people in the area. Two ICUs participated in this study. One ICU was an 8-bed unit, accommodating emergency patients with acute medical or surgical diseases and treating approximately 600 patients annually. The other ICU was a 10-bed unit, admitting those who underwent elective surgery and those who deteriorated after hospitalization on general wards and treating approximately 900 patients annually. The participants in this study worked at one of these ICUs exclusively.

### Participants

HCWs who met the following criteria were included: 1) those who worked exclusively at either ICU; and 2) those who agreed to respond to the questionnaire survey and submit their smartphones for microbial investigation. HCWs who did not possess a smartphone were excluded. No restrictions as to the presence or absence of lacerations on the touchscreens or posterior surfaces of the smartphones were placed.

### Measurements

This study included two investigations: a questionnaire survey and a microbiological investigation of participants’ smartphones. The questionnaire asked about participants’ characteristics (age, sex, and profession), information regarding smartphone use (duration of ownership of the present smartphone, the presence of a cover over the smartphone, regular disinfection of the smartphone, and the use of film over the touchscreen), and awareness of bacterial contamination associated with smartphones. The duration of ownership of the present smartphone was defined as the duration from the purchase of the smartphone to the date of the survey. Regular disinfection was defined as having the habit of cleaning the smartphone surfaces with disinfectants such as alcohol-containing materials, irrespective of the cleaning frequency. Participants were considered to have awareness of bacterial contamination associated with mobile phones when they recognized that mobile phones could be a fomite of bacteria potentially causing nosocomial infections.

The microbiological investigation of smartphones proceeded as follows [22]. A sterile cotton swab was immersed in saline solution (0.9% NaCl) and immediately rubbed over the touchscreen or posterior surface of the smartphone. When the posterior surface of a smartphone was covered or contained in cases, the cover or case was removed, and the posterior surface was rubbed directly. This swab was immediately rolled and spread over a sheep blood agar plate, which was incubated at 37 °C for 48 h under aerobic conditions. Subsequently, the number of colonies that grew on the plate was counted. MALDI Biotyper (Bruker Daltonics GmbH, Bremen, Germany) was used with the manufacturer-provided database for bacterial identification. Drug susceptibility of a microorganism was judged based on clinical breakpoints set by the Clinical and Laboratory Standards Institute [23]. In particular, isolates suspected to be *Staphylococcus aureus* were tested for cefoxitin resistance (30-μg discs, MASTDISCS) as a surrogate marker for the detection of methicillin resistance based on the disk diffusion method [24].

Participants were approached without advance announcement of this study so that they could not clean their smartphones before the microbial investigation.

### Statistical analysis

Descriptive statistics were used to describe the prevalence of microbial contamination on each surface of the smartphones and the type of associated bacterial species as the primary outcomes. Fisher’s exact test was used to compare dichotomous variables. Continuous variables are shown as medians with interquartile ranges (IQRs). A multivariable logistic regression analysis was also conducted to identify factors related to bacterial contamination of the posterior surfaces of smartphones. Fully adjusted models included three variables that were chosen a priori: female sex, use of a cover, and regular disinfection of smartphones. Stata SE version 15.1 (StataCorp, College Station, TX, USA) was used for statistical analysis.

This study was approved by the institutional review board at Kurashiki Central Hospital. This study was conducted in accordance with the relevant guidelines and regulations. Informed consent was obtained from individual participants.

## Results

### Characteristics of study participants

A total of 90 HCWs in the ICUs were approached, and 6 HCWs were excluded because they did not have smartphones. Finally, 84 consecutive participants who had smartphones were included in this study (Table 1). Their median age was 31 years, and 53 (63.1%) HCWs were female. Nineteen (22.6%) HCWs were physicians, 49 (58.3) were nurses, 9 (10.8%) were rehabilitation therapists, and the remaining participants included an assistant, clerks, and a pharmacist.

**Table 1:** Characteristics of Study Participants and Smartphone Habits

Characteristics	
No. of participants	84
Median age (IQR)	31 (28–38)
Age category, n (%)	
≤ 29	32 (38.1%)
30–39	27 (44.0%)
40–49	11 (13.1%)
50–59	2 (2.4%)
≥ 60	2 (2.4%)
Female, n (%)	53 (63.1%)
Type of profession, n (%)	
Physician	19 (22.6%)
Nurse	49 (58.3%)
Rehabilitation therapist	9 (10.7%)
Pharmacist	1 (1.2%)
Clerks	5 (6.0%)
Assistant	1 (1.2%)
Using smartphones at bedside, n (%)	23 (27.4%)
Frequency of using smartphones, n (%)	
1–4 times/day	10 (11.9%)
5–9 times/day	28 (33.3%)
10–14 times/day	11 (13.1%)
≥ 15 times/day	35 (41.7%)
Places of most frequent smartphone use, n (%)	
Home	80 (95.2%)
Resting room at the hospital	2 (2.4%)
Bedside	1 (1.2%)
Others	1 (1.2%)
Regular disinfection of smartphone, n (%)	9 (10.7%)
Washing hands before using smartphones, n (%)	5 (6.0%)
Washing hands after using smartphones, n (%)	4 (4.8%)
Characteristics of smartphones	
Median duration of the current smartphone use (IQR) (month)	18 (12–29)
Use of a film over the touchscreen	66 (78.6%)
Use of a cover	69 (82.1%)

*Abbreviation*: *IQR* interquartile range

### Questionnaire survey on smartphone use

The current smartphone of each participant was owned for a median of 18 months (IQR, 12–29 months) (Table 1). Sixty-six (78.6%) participants placed a film on the touchscreen, and 69 (82.1%) used a cover on the posterior surface of the smartphone. Twenty-three (27.4%) participants used their smartphones at the patient bedside.

Only 9 (10.7%) participants reported that they regularly sanitized their smartphones. They all used alcohol to clean their smartphones, irrespective of cleaning frequency. Five (6.0%) and four (4.8%) participants washed their hands before and after they used their smartphones, respectively. Seventy (83.3%) participants recognized that mobile phones could be a fomite of bacteria potentially causing nosocomial infections.

### Microbiological investigation

Forty-nine smartphones (58.3%; 95% confidence interval [CI], 45.8 to 67.6%) were contaminated with bacteria; the touchscreen was contaminated in 27 (32.1%), posterior surface in 39 (46.4%), and both surfaces in 17 (20.2%) smartphones. The posterior surface was more frequently contaminated than the touchscreen (*p* = 0.041).

Cultures from the touchscreen of 27 smartphones grew a median of 2 colonies (IQR, 1–6 colonies). A single species was isolated from 18, two species from 6, and three or more species from 3 smartphones. Coagulase-negative staphylococci (CNS) were the most frequently cultured bacteria (21 smartphones), followed by *Bacillus* species (13 smartphones) (Table 2). *S. aureus,* which was methicillin-resistant, was isolated from only one smartphone (1.1%). There was no significant difference in the frequency of touchscreen contamination by the use of film over the touchscreen (*p* = 0.26), sex (*p* = 0.64), regular disinfection of smartphones (*p* = 0.46), or handwashing before smartphone use (*p* = 0.66).

**Table 2:** Bacteria Species Detected on Each Smartphone Side

Organisms	Touchscreen			Posterior Surface		
	Overall (*n* = 84)	With a film (*n* = 66)	Without a film (*n* = 18)	Overall (*n* = 84)	With a cover (*n* = 69)	Without a cover (*n* = 15)
Gram-positive bacteria						
*Coagulase-negative staphylococci*	21 (25.0%)	14 (21.2%)	7 (38.9%)	22 (26.2%)	15 (21.7%)	7 (46.7%)
*Bacillus spp.*	13 (15.5%)	10 (15.2%)	3 (16.7%)	26 (31.0%)	19 (27.5%)	7 (46.7%)
*α-Streptococcus*	1 (1.2%)	1 (1.5%)	–	–	–	–
*Actinomyces oris*	1 (1.2%)	1 (1.5%)	–	–	–	–
*Micrococcus sp.*	–	–	–	1 (1.2%)	1 (1.4%)	–
*Rothia mucilginosa*	1 (1.2%)	–	1 (5.6%)	–	–	–
*Staphylococcus aureus*	1 (1.2%)	–	1 (5.6%)	–	–	–
Gram-negative bacteria						
*Acinetobacter lwoffii*	–	–	–	1 (1.2%)	1 (1.4%)	–
*Enterobacter cloacae*	–	–	–	1 (1.2%)	1 (1.4%)	–
*Pseudomonas fulva*	–	–	–	1 (1.2%)	1 (1.4%)	–
*Roseomonas mucosa*	–	–	–	1 (1.2%)	1 (1.4%)	–

Cultures from the posterior surface of 39 smartphones grew a median of 2 colonies (IQR, 1–3 colonies). A single species was isolated from 32, two species from 2, and three or more species from 5 smartphones. *Bacillus* species were the most frequently cultured (26 smartphones), followed by CNS (22 smartphones) (Table 2). There was no significant difference in the frequency of contamination of the posterior surface by cover use (*p* = 0.58), sex (*p* = 0.82), regular disinfection (*p* = 0.49), or handwashing before smartphone use (*p* = 0.66). Multivariable logistic regression analysis showed that female sex (odds ratio [OR] 0.86; 95% CI, 0.35–2.10; *p* = 0.73), cover on the posterior surface (OR 0.73; 95% CI, 0.24–2.24; *p* = 0.58), and regular disinfection of smartphones (OR 0.55; 95% CI, 0.13–2.37; *p* = 0.42) were not associated with bacterial contamination of the posterior surface (Hosmer-Lemeshow *p* = 0.59) (Table 3).

**Table 3:** Results of the multivariable logistic regression analysis

Variable	Odds ratio	95% Confidence Interval	*P*-value
Female	0.86	0.35–2.10	0.73
Cover on the posterior surface	0.73	0.24–2.24	0.58
Regular disinfection	0.55	0.13–2.37	0.42

## Discussion

The present study showed that 58.3% of smartphones used by Japanese ICU HCWs were contaminated with bacteria, with 46.4% of the posterior surfaces being involved. CNS and *Bacillus* species were frequently isolated from these smartphones. No factor was found to be associated with bacterial contamination of the posterior surface.

Previous studies suggested that 10–100% of mobile phones in healthcare settings could be contaminated [11–13]. Most of these studies focused on conventional keypad mobile phones. However, a limited number of studies focused on smartphones and suggested that 20.9–99.2% of them in healthcare settings are contaminated [21, 25–28]. Common microorganisms associated with mobile phone contamination overall included *S. aureus*, CNS, and *Bacillus* species [11–13], consistent with the present study findings. Although antimicrobial-resistant bacteria were isolated from 1.1% of smartphones, *S. aureus*, CNS, and *Bacillus* species can also be causative agents of nosocomial infections, and thus caution regarding smartphone microbial contamination is needed.

In the present study, 46.4% of posterior surfaces of smartphones were contaminated with bacteria, significantly more frequently than touchscreens. The numbers of colonies on touchscreens and posterior surfaces of the smartphones were similar, though small. One previous study screened both surfaces of smartphones, but did not differentiate the surfaces with respect to the prevalence of bacterial contamination [26]. To the best of our knowledge, the present study is the first to focus particularly on the posterior surface of smartphones. In the present study, whereas the touchscreen was contaminated only with Gram-positive bacteria, the posterior surface of the smartphones was contaminated with both Gram-positive and Gram-negative bacteria. The reason for this difference is uncertain.

We considered the following hypotheses for factors related to bacterial contamination of the posterior surfaces of smartphones. First, existing evidence is conflicting regarding whether sex might be a factor associated with smartphone bacterial contamination [29]. Some studies suggested that women store their smartphones in bags, which are associated with high bacterial levels, and heat generated by smartphones and the inside area of bags could support bacterial growth [14, 30]. Thus, female sex was set as a potential factor associated with smartphone bacterial contamination. Second, the posterior surface of smartphones, once covered, or the inside area of the cover is less frequently cleaned by users than the touchscreen, and bacteria are expected to remain on the surface. Third, regular disinfection of smartphones could also reduce or prevent bacterial colonization on smartphone surfaces. However, none of these were found to be associated with bacterial contamination of the posterior surface.

The results of the present study suggested that the posterior surface was more frequently contaminated than the touchscreen, regardless of whether it was covered. Although the posterior surface is not as frequently touched as the touchscreen, it could be a fomite of transmitting bacteria to the healthcare environment once contaminated. Thus, we recommend that the posterior surface of smartphones be cleaned, similar to the touchscreen, in order to avoid cross-contamination of healthcare settings, particularly ICU settings.

In response to the COVID-19 pandemic, the United States Centers for Disease Control and Prevention (CDC) recommends the use of wipeable covers for electronics [31]. However, once contaminated, the area under the cover can harbor pathogenic bacteria that can cause nosocomial infection. Furthermore, if the cover of a smartphone is incidentally removed, bacteria under the cover may move to the healthcare or home environment. The present study suggested that the posterior surfaces, whether or not they were covered, were similarly contaminated with bacteria. Thus, the posterior surfaces need to be cleaned regardless of the presence of a cover.

Whereas the United States CDC recommends that users of electronic devices follow the manufacturer’s instructions for cleaning and disinfecting products, they also recommend the use of alcohol-based wipes or sprays containing at least 70% alcohol to disinfect the touchscreen [31]. Previous studies have demonstrated that a silver-containing antibacterial coating, light-activated antimicrobial agents, antibacterial wipes containing alcohol, chlorhexidine, chlorine dioxide, or quaternary ammonium compounds, and ultraviolet light irradiation successfully decontaminate the touchscreen, some of which also effectively decontaminate the posterior surface [32]. Thus, the posterior surface of smartphones should be cleaned using such options. However, it is unclear how often the posterior surface of smartphones needs to be cleaned, for which further studies are awaited.

The present study had some limitations. First, it had a small sample size and a single infection control culture, which might limit the generalizability of the findings. Studies to confirm the present findings are needed. The small sample size might also have been responsible for the lack of statistical significance of potential factors associated with bacterial contamination of the posterior surface. Second, the Hawthorne effect might have led to advanced cleaning of smartphones by participants with knowledge of this study. However, the impact of this bias may be limited by the unannounced microbial investigation of the smartphones and completion of this study in a short period. Third, smartphones were checked only for bacterial contamination. While clinicians during the present COVID-19 pandemic may be interested in viral contamination of smartphones, no measures to examine viral contamination were available for this study. However, the fact that the posterior surfaces of smartphones were more frequently contaminated with bacteria than touchscreens, regardless of the use of covers, will make us cautious about viral contamination of smartphones as well.

## Conclusions

The results of the present study suggested that approximately 60% of smartphones used by HCWs were contaminated with bacteria, and the posterior surface of tested smartphones were more significantly contaminated than the touchscreen. CNS and *Bacillus* species were frequently isolated from these smartphones. Regardless of the use of a cover on a smartphone, the posterior surface, as well as the touchscreen, should be routinely cleaned to prevent potential bacterial dissemination in healthcare settings.

## Data Availability

All data generated or analysed during this study are included in this published article.
